# “It was a foregone conclusion”: a qualitative study of women’s experiences and meaning-making of later-in-life abortion in Belgium

**DOI:** 10.1080/26410397.2024.2444719

**Published:** 2025-01-06

**Authors:** Kato Verghote, Nathalie Neeser, Tenzin Wangmo, Guido Pennings, Veerle Provoost

**Affiliations:** a Ph.D. Student, Bioethics Institute Ghent; Department of Philosophy and Moral Sciences, Ghent University, Ghent, Belgium.; b Ph.D. Student, Institute for Biomedical Ethics, University of Basel, Basel, Switzerland; c Senior Researcher, Institute for Biomedical Ethics, University of Basel, Basel, Switzerland; d Professor, Bioethics Institute Ghent, Department of Philosophy and Moral Sciences, Ghent University, Ghent, Belgium

**Keywords:** abortion, age, decision-making, social norms, qualitative research

## Abstract

Abortion is an indispensable healthcare service for women of all reproductive ages. Research on abortion is often focused on younger women, neglecting those who are closer to the end of their reproductive lifespan. This study presents findings from qualitative interviews with Belgian women who had an abortion at the age of 40 or older, conducted between May 2022 and April 2023. Using interpretative phenomenological analysis, we explored the experiences and decision-making processes of women who had abortions later in life. We identified three main themes. First, the women’s families were at the centre of their abortion decisions, with some women also presenting advanced age as a significant factor in their decision-making. This emphasis on age was connected to perceived social norms about appropriately timed childbearing. Second, the women experienced their unplanned pregnancies as both physically and emotionally demanding, and desired to terminate them as soon as possible. Some participants felt additional emotional burdens because of delays caused by the mandatory waiting period and/or busy schedules at abortion centres. Third, the women expressed feelings of self-blame for their unplanned pregnancies. This self-blame was closely tied to their expectation of social disapproval, which made them cautious to share their abortion experience with people in their social circle. This study enhances our understanding of the experiences and meaning-making of abortion in women of advanced reproductive age. It highlights the need to destigmatise the topic and the importance for professionals and researchers to consider family preservation and advanced reproductive age as potential factors shaping abortion decision-making.

## Plain Language Summary

Abortion is a crucial healthcare service for women of all ages. However, research on abortion often focuses on younger women. Therefore, we do not know much about the abortion experiences of those who are of more advanced reproductive age. For this study, we have conducted interviews with eight Belgian women who had an abortion at the age of 40 or older. These interviews allowed us to explore in depth the women’s experiences and motivations to have an abortion at this stage in their lives. Based on their stories, we identified three main themes. First, we describe how the women’s abortion decisions centred around their families. Some participants also considered their older age an important reason to have the abortion. Second, we explain that our participants described their unplanned pregnancies as physically and emotionally tough. This was the reason why they all wanted to have the abortion as soon as possible. In the third theme, we discuss how the women blamed themselves for becoming pregnant unplanned. They also said that they expected to receive negative comments from people in their social circle. Thus, most participants decided to remain silent about their abortion experience. We want to share these findings with research communities, Belgian and international abortion centres, and individuals interested in the topic. This way, we hope to contribute to an open debate about abortion, and the unique circumstances in which people make abortion decisions.

## Résumé

L’avortement est un service de santé indispensable pour les femmes de tous âges reproductifs. La recherche sur l’avortement se concentre souvent sur les jeunes femmes et elle néglige celles qui sont plus proches de la fin de la période au cours de laquelle elles sont en âge de procréer. Cette étude présente les résultats d’entretiens qualitatifs menés auprès de femmes belges ayant avorté à l’âge de 40 ans ou plus. À l’aide de l’analyse phénoménologique interprétative, nous avons étudié l’expérience et le processus de prise de décision des femmes ayant avorté plus tard dans leur vie. Nous avons identifié trois thèmes principaux. Premièrement, la famille des femmes était au centre de leur décision d’avorter, certaines femmes présentant aussi l’âge avancé comme un facteur important dans leur prise de décision. Cet accent placé sur l’âge était lié aux normes sociales perçues concernant le moment approprié de la maternité. Deuxièmement, les femmes considéraient leurs grossesses non planifiées comme exigeantes à la fois physiquement et émotionnellement, et elles souhaitaient les interrompre le plus tôt possible. Certaines participantes ont ressenti une charge émotionnelle supplémentaire en raison des retards causés par la période d’attente obligatoire et/ou l’emploi du temps chargé des centres d’avortement. Troisièmement, les femmes ont exprimé un sentiment de culpabilité pour leur grossesse non planifiée. Cette culpabilité était étroitement liée à leur attente de désapprobation sociale, ce qui les incitait à se montrer prudentes avant de partager leur expérience de l’avortement avec des personnes de leur cercle social. Cette étude améliore notre compréhension des expériences et de la construction de sens de l’avortement chez les femmes d’un âge reproductif avancé. Elle souligne la nécessité de déstigmatiser le sujet et l’importance pour les professionnels et les chercheurs de tenir compte de la protection de la famille et de l’âge reproductif avancé comme des facteurs potentiels influant sur la prise de décision en matière d’avortement.

## Resumen

El aborto es un servicio de salud indispensable para las mujeres en cualquier edad reproductiva. Las investigaciones sobre aborto a menudo se centran en mujeres más jóvenes y hacen caso omiso de aquéllas que se acercan al final de su vida reproductiva. Este estudio presenta los hallazgos de entrevistas cualitativas con mujeres belgas que tuvieron un aborto a los 40 años o más. Utilizando el Análisis Fenomenológico Interpretativo, exploramos las experiencias y el proceso de toma de decisiones de mujeres que tuvieron un aborto a una edad más avanzada. Identificamos tres temas principales. Primero, la familia de cada mujer figuraba al centro de sus decisiones sobre el aborto; algunas mujeres también mencionaron su edad avanzada como un factor significativo en su toma de decisiones. Este énfasis en la edad estaba conectado con la percepción de las normas sociales sobre el momento adecuado para tener hijos. Segundo, la experiencia de las mujeres con su embarazo no planificado fue física y emocionalmente agotadora, y ellas deseaban interrumpir su embarazo lo antes posible. Algunas participantes sentían una carga emocional adicional debido a los retrasos causados por el período de espera obligatorio y/o a horarios ajetreados en los centros de aborto. Tercero, las mujeres se culpaban a sí mismas por su embarazo no planificado. Este sentimiento de culpa estaba vinculado estrechamente con su expectativa de desaprobación social, por lo que eran cautelosas en cuanto a revelar su experiencia de aborto a personas en su círculo social. Este estudio mejora nuestra comprensión de las experiencias y el dar sentido al aborto en mujeres en edad reproductiva avanzada. Hace hincapié en la necesidad de desestigmatizar el tema y en la importancia de que profesionales e investigadores consideren la preservación de la familia y la edad reproductiva avanzada como posibles factores que inciden en la toma de decisiones sobre el aborto.

## Introduction

Worldwide, there are 35 abortions per 1000 women* of reproductive age each year.^1^ In Belgium, approximately one in five women has an abortion at some point in her life,^2^ and in both the United Kingdom and the United States the numbers are higher, with estimates suggesting that roughly one in three women have an abortion by the age of 45.^3,4^ Although a wealth of research has been conducted to gain insight into women’s experiences and motivations to have an abortion, most of these studies have focused on the experiences of teenagers or young adults.^5,6^ Women who are closer to the end of their reproductive lifespan are rarely the focus of research on abortion, and when they are included in studies, they are often part of larger research samples that include women of all reproductive ages.^7,8^ Consequently, these studies do not typically consider the experiences of abortion in women of advanced reproductive age in detail. Moreover, research on women’s motivations for abortion often addresses reasons that are commonly associated with a woman’s young age, such as not being emotionally ready to have a child yet, relationship instability, financial constraints and the desire to explore life to the fullest before embarking on motherhood,^9,10^ but which may not be exclusive to younger women. Empirical studies with women of varying ages who had an abortion found that relationship instability and demanding work conditions were key factors motivating the decision for abortion among older women,^11^ and a particular line of reasoning was identified in participants who were mothers: they sought to protect the needs of their children and to maintain the size of their family as they considered it to be complete.^12–14^ Empirical research that specifically examines advanced reproductive age as a factor potentially guiding women’s abortion decision-making is scarce, and older reproductive age as such has not been the focus of any recent qualitative study on abortion.

Although it is commonly assumed that most abortions are undergone by young women and girls,^15^ research by Sedgh and colleagues^16^ shows that women in their mid-reproductive years (between 25 and 35) have the highest proportion of abortions. Registration data from Belgium demonstrate that, in 2021, the average age of women who had an abortion was 29, and over 6% of all abortions occurred in women aged 40 or older.^2^ Similar, or slightly higher, ages have been found in the United States,^17^ and in other European countries, such as the United Kingdom and Portugal.^18,19^ Women’s reproductive timing and behaviour are subject to regulation and public scrutiny, as well as criticism.^20^ Furthermore, the stigmatisation of advanced age motherhood has been repeatedly documented in empirical research.^21,22^ Hence, women’s reproductive decisions (whether to continue or terminate a particular pregnancy) and their age need to be understood in the context of societal norms and attitudes. Despite ongoing debates about what constitutes advanced age in reproduction and parenthood, contemporary research and demographic reports increasingly employ 40 as a cut-off age to indicate more advanced age categories.^22–24^ In alignment with this rationale, we decided to define later-in-life abortion as having had an abortion at the age of 40 or older. Given the limited literature on later-in-life abortion, the aim of this study was to address the following research questions: How do Belgian women experience their decision-making in the context of a later-in-life abortion, how do they make meaning of the abortion, and what role, if any, does age play in these processes? This study supports the notion that abortion should be regarded as a sexual and reproductive right, enabling all individuals to decide whether, when and by what means to have a child or children, and how many children to have, and to have access over their lifetimes to the information, resources, services and support necessary to achieve all of the above, free from discrimination, coercion, exploitation and violence.^25^

The contribution of empirical research on this topic may facilitate improvements in reproductive healthcare and the endorsement of abortion as an indispensable sexual and reproductive right regardless of age.

## Background

In Belgium, access to abortion has been legally permitted since 1990^26^ and has been removed from the country’s criminal code through a separate law in 2018.^27^ Abortion on request (no justification or grounds must be provided) is permitted up to the twelfth week of pregnancy. After that, abortion is only allowed in instances where the pregnancy endangers the woman’s health or when it is established that the child will be born with an incurable disease. Women who are less than seven weeks pregnant are offered a choice between medical abortion (i.e. intake of medications) and surgical abortion (i.e. suction curettage). Between the seventh and twelfth week of gestation, only the latter abortion method is offered.

In Flanders (Dutch-speaking northern part of Belgium), most abortions are performed in specialised centres (the majority of which belong to the so-called “LUNA abortion centres”).^28^ There are six centres in total, spread across the Flemish provinces, with an additional centre in Brussels (Brussels is not a part of Flanders). In Wallonia (French-speaking southern part of Belgium), abortion is part of a wide range of services offered by “family and marriage planning centres”. In addition to abortion, these centres offer services such as sex education, contraception, screening of sexually transmitted infections, and information on family law provisions (e.g. regarding divorce, adoption, inheritance).^29^ There are 72 of these centres in Wallonia, which means that, on average, women living in Flanders must travel longer and further (i.e. 50–60 km at most) to visit an abortion centre compared to women living in Wallonia. The disparate operational approaches of the centres are firmly embedded in the social and political structures that existed in these two geographical regions prior to the 1990s. (For an overview of Belgium’s history of abortion care see De Kort.)^30^ Due to budget constraints (that restricted the possibility of involving French-speaking researchers and the translation of interview transcripts), the geographical scope of this study was limited to Flanders.

The abortion procedure in Flanders requires two separate private appointments (additional visits are optional). During the first appointment, the centre arranges a meeting with a psychosocial counsellor. During this meeting, individuals seeking an abortion (and, if they so wish, an accompanying confidant) have the opportunity to discuss their reasons for seeking an abortion and to address any remaining questions or doubts. This is followed by a mandatory waiting period of 6 calendar days.^†^ If the initial decision to terminate the pregnancy is reaffirmed, a second appointment is scheduled to proceed with the abortion. At present, those seeking an abortion are required to pay €4.5 for the procedure at an accredited centre.^2^ The remaining costs of €556.5 are covered by health insurance (which is mandatory in Belgium).

## Methods

### Participant recruitment

The first author became acquainted with the LUNA abortion centres through repeated contact with staff members and visits to two different centres, during which she introduced the study to the staff. During the women’s first or second appointment, staff members informed eligible women (≥40 years at the time of the abortion) about the study and provided the researchers’ contact details (e-mail and telephone) if anyone was interested in participating. Six women were recruited through staff referral, one woman responded to a call on the abortion centres’ social media, and one additional participant was recruited through a word-of-mouth strategy. Prior to the commencement of the study, the interviewer was not personally acquainted with any of the participants. The study sample is in line with other studies applying interpretative phenomenological analysis (IPA), as the intent was to identify rich and detailed accounts of the participants’ lived experiences^31^ and not to attain a sample size that would result in data “saturation”.^32^

### Interviews

The first author conducted all interviews between May 2022 and April 2023. The participants were free to choose both time and location of the interview. All interviews were conducted as one-time one-on-one interviews at the participants’ homes. Written informed consent (for conducting the interviews and publication based on these interviews) was obtained before each interview. Participants were informed that the interviewer was a PhD student with an interest in their experiences of having an abortion at the age of 40 or over. An interview guide was designed as a basis, covering four main topics: (i) the women’s mothering experiences (if applicable), (ii) their decision-making process regarding their later-in-life abortion, (iii) their experiences and meaning-making of the later-in-life abortion and the time afterwards and (iv) social reactions and support. The interview questions were distributed to LUNA staff prior to the interviews, and their feedback was incorporated into the final version of the interview guide. Throughout the interviews, the participants were assigned the role of experts^33^ as the interviewer positioned herself as a woman without personal experience of abortion, without children and someone who was visibly younger than the participants. Additionally, the interviewer maintained a non-judgmental demeanour to encourage participants to freely share their experiences,^34^ and she emphasised that there were no right or wrong answers to the interview questions. The first author acknowledges her role in co-constructing the data, for instance by framing the interview questions and responding to the participants’ answers.

Field notes were taken during and after the interviews to reflect on the course of the interview. The interviews were audio recorded and transcribed verbatim. All data that could (in)directly lead to the identification of the participants or their family members were replaced by pseudonyms. Participants were not asked to check their transcripts, nor were they engaged in the process of data analysis. The study was approved by the Ethics Committee of the Faculty of Arts and Philosophy, Ghent University on 15 March 2022 (Ref. 2022-4).

### Data analysis

Given our aim to analyse the lived experiences and meaning-making of the participants in depth, we guided our study design and analysis by IPA.^33^ IPA requires an inductive, participant-oriented approach^35^ as researchers practice a “double hermeneutic”,^36^ trying to make sense of how the participants made sense of the phenomena they have experienced in their immediate personal and social worlds. Through in-depth exploration of participant experiences, we attempted to gain an insider’s perspective.^37^ In line with IPA, our analysis combines participant descriptions (accompanied by quotes to explain our findings and show that the analysis is grounded in the data) with interpretive comments by the researchers.^31^

The first step in the current analysis involved repetitive reading of the transcripts while keeping track of initial observations and reflections through memo-writing. In addition to the literal content of the data, attention was paid to how participants had expressed themselves. In a second phase, using qualitative digital software (NVivo), the first author engaged in an iterative process of searching for patterns in the data, constructing codes and clustering codes into preliminary themes. Throughout the analysis, seven auditing meetings^38^ were held with the co-authors (though not all attended every meeting). During these sessions, the first author presented selected excerpts of illustrative data, which were discussed and used to challenge and refine tentative theme structures. Interdisciplinary interpretation of the data was encouraged through verbalisation of the authors’ insights in relation to their respective academic backgrounds (i.e. philosophy, gerontology, social anthropology and gender studies). These auditing meetings enabled the first author to strengthen the depth and rigour of the final thematic structure.

## Results

We obtained a sample of eight women who had had an abortion at the age of 40 or older. All participants were white and born in Belgium. Seven of the participants were married or in a committed relationship when they became pregnant later in life. One participant became pregnant later in life while casually dating. All women had one or several children, were in paid employment and had completed secondary education (*n* = 2), a bachelor’s degree (*n* = 3) or a master’s degree (*n* = 3). The women’s religious affiliation varied: three identified as Catholic and five said they were not religious or clarified that they did not identify with any specific religion. The age at which the participants had a later-in-life abortion ranged between 40 and 45 years. Seven participants chose to have a surgical abortion and one opted for a medical abortion. Most of the women were interviewed a few weeks after their abortions, while two were interviewed one to two years later. At the time of the interview, three participants had had one or two previous abortions. None of the women who consented to take part in the study subsequently withdrew their participation. See Table 1 for an overview of the main characteristics of the participants. The interviews lasted between 49 and 107 minutes (with an average of 74 minutes).

**Table 1.** Participant characteristics

Pseudonym	Age at LILA^a^	Method of LILA^a^	Number of children	Children’s ages^b^	Relationship status at LILA^a^
Angelique	43	Surgical	2	1–6	Married/domestic partnership
Christina	42	Surgical	2	13–18	Single
Gaelle	42	Medical	1	1–6	LAT^c^
Irene	43	Surgical	2	13–18	Married/domestic partnership
Iris	40	Surgical	2	7–12	Married/domestic partnership
Juliet	42	Surgical	2	7–12	Married/domestic partnership
Louisa	42	Surgical	1	7–12	Married/domestic partnership
Naomi	45	Surgical	4	13–24	LAT^c^

^a^ Later-in-life abortion.

^b^ The participants’ children’s ages at the time of the later-in-life abortion are presented as age categories so as to minimise the chances of identifiability of the participants and their unique family composition.

^c^ Living apart together - a committed relationship in which partners choose to maintain separate living spaces.

**Figure 1.**
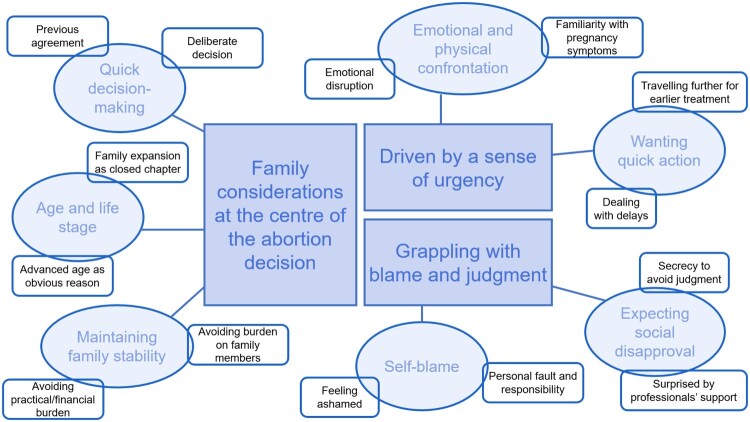
Visual representation of themes and codes

We identified three themes (see Figure 1). The first theme relates to the participants’ decision-making process about opting for abortion later in life. We describe two main reasonings found in their decisions: age and life phase and maintaining the status quo of the family. The second theme explores how the unplanned pregnancies created a sense of urgency that made the women want to act as quickly as possible. The final theme examines the self-blame and shame that the women experienced in relation to their unplanned pregnancy and abortion, and how these feelings were sometimes intensified due to their belief that they should have known better at their age. We discuss how this is associated with expectations of social judgement, which kept most women silent about their abortion experience.

### Family considerations at the centre of the abortion decision

Early on in the interview, each participant described how obvious it was for her (and her partner) to terminate the later-in-life-pregnancy. The women portrayed their decision as “clear” or “a foregone conclusion”, requiring minimal thought or contemplation of other options. Most of the participants framed the decision as predetermined, as they had previously discussed the possibility of another pregnancy with their partner and had mutually agreed to opt for abortion in such a scenario. This previous agreement “*was not questioned again”* (Gaelle), or was quickly reaffirmed:

*“And that was just, yes [reconstructs conversation with partner after finding out she was pregnant]: ‘This is no big deal, right? Are we going for an abortion?’, ‘Yes, we always agreed on that, right?’, ‘Ah yes, that’s no problem’. I called [the abortion centre] immediately. And all of that in twenty minutes of time while we were having coffee at the kitchen table.”* (Louisa)

Although the decision did not take much time, most participants described their decision-making process as “deliberate”. They offered two main reasonings for their decision. The first one related to their age and life stage. The women discussed a feeling of inappropriateness concerning the expansion of their family at this time in their lives. They expressed that having another child did “not fit” their present family situation and personal lifestyle. Two participants remarked that life could be perceived as consisting of various “chapters” or “phases”. As they were in their forties, they considered themselves to no longer be in the appropriate chapter or phase to have another child: “*That phase of having children is just over”*, said Irene. Or as Naomi put it: “*That was a closed chapter”*. Four participants spontaneously referred to their advanced age as a reason not to further expand their family:

*“We [Louisa and her partner] are at an age now that it is not really … it cannot … Or ‘can’ is said too much. No. It does not fit, it is not ideal. We are already … He is 47. Well, he was 45 at the time, and I was 42. It did not occur to me to get pregnant at that age either. I felt like those celebrities.”* (Louisa)

Most of these women introduced advanced age as an *obvious* reason justifying their decision for abortion, drawing on the assumption that such reasoning was in line with social norms about appropriately timed motherhood and family building. As Irene clarified, she had the abortion now (in her forties) for much the same reasons as when she previously had an abortion in her thirties, “*although now it was easy: ‘I am 40+! Come on!’”*. However, despite having been fully informed about the study’s subject (i.e. abortion at age 40 or older) in advance, half of the participants did not spontaneously cite their age as a reason for choosing abortion during the interviews. Instead, they discussed factors unrelated to age. The women who mentioned advanced age as a contributing factor in their decision-making consistently cited other reasons that they deemed equally or more important.

Further exploration into the factors behind the women’s decisions to terminate their later-in-life pregnancies revealed a second type of reasoning: the participants considered abortion a means to maintain their existing family structures and situations. Although some women indicated that their desired family size was complete (*“I don’t want more than two children”* – Juliet), for most participants, the desire to maintain the status quo of the family was primarily expressed in connection with a fear of potential consequences of expanding their family at this point in their lives. Several women reflected on the meaning of continuing with their pregnancy and anticipated their family to be in a difficult financial and practical (i.e. in terms of time and physical space within their homes) situation:

*“I immediately felt like: ‘I cannot have that, we [participant and partner] don’t live together, it’s practically not feasible, financially it would be much too heavy, it would make everything so much more complex’. That’s what I thought. It really was not an option at this moment.”* (Gaelle)

Moreover, the women expressed concern that having another child could potentially put a strain on family members and on their mutual relationships. Some described how adding a new member to the family could adversely affect their children. Gaelle, for instance, clarified that her daughter was still adjusting to her mother’s new romantic relationship, and Iris indicated that her son was going through an emotionally challenging time. Therefore, these women reasoned that the addition of a new sibling would increasingly burden their children. A few participants believed that family expansion at this time in their lives would impair their romantic relationships with their partners. Irene recalled a time of strain in her relationship with her partner when they had their two children over a decade ago. Having an abortion was a means for her to avoid facing similar relationship issues in the future:

*“I will be honest about that; that period of having younger kids was not the best period for our relationship. And I think if I get back into that process [of having young children] now, then there is a very good chance that we will go through a dip in our relationship, and that my partner would say: ‘This is what you have wanted’. And I want to … (…) I think I have to shift my priorities to the relationship instead of raising children again.”* (Irene)

Finally, two participants voiced concerns about family expansion because of possible personal harm it might bring. Naomi mentioned a present medical condition which may entail dangers during pregnancy and childbirth. Simultaneously, she associated this with her responsibility towards her older children:

*“If something were to happen during childbirth, I can’t do that to my other children, like … leaving them behind just because I want to take the risk of bringing another baby into the world.”* (Naomi)

Louisa discussed her past experience with postnatal depression and feared the potential of relapse if she were to give birth again. She, like Naomi, considered the burden this would put on her partner and child. Being afraid that “*it would all end up worse”* (Louisa), the women decided to have an abortion as they believed it was the best course of action to maintain family stability. Having another child at this point in their lives “*would not have been a good move for anyone”*, as Gaelle put it.

### Driven by a sense of urgency

Although all participants presented their decision to have an abortion as straightforward, this did not mean that the women were not emotionally affected. All participants described the period between discovering their pregnancy and having the abortion as emotionally disruptive, or “*a drain on your life”*, as Iris said. A few participants pointed to being consistently confronted by their pregnant bodies, as they experienced sickness, nausea, and fatigue. Moreover, having previously experienced full-term pregnancies, many of the women expressed a profound understanding of what was happening to their bodies. They recognised the onset of pregnancy at an early stage, given their familiarity with the associated symptoms. This realisation intensified the women’s feelings of finding themselves in an urgent situation: “*I don’t want to take this lightly. I understand that if I don’t do anything, I’ll have a child in nine months. That is why I acted very quickly”* (Irene). Accordingly, all women expressed a strong desire to terminate their pregnancies as soon as possible:

*“I felt incredibly nauseous. You feel the whole thing [the pregnancy] happening. You want it gone as soon as possible, to put it bluntly. I also wanted to get rid of that feeling of being pregnant.”* (Angelique)

*“I felt it immediately. I had the same thing with my previous pregnancies. I also immediately felt my breasts tighten. And my appetite, I was incredibly hungry. I immediately had a déjà vu to my previous pregnancies. I thought: ‘I’m not going to let that be and wait around.’”* (Christina)

Though some participants recounted being able to promptly secure an appointment at the abortion centre, five women expressed disappointment at their inability to schedule an appointment earlier. Naomi and Iris felt that the mandatory waiting period was unnecessary for them:

*“And I was like: ‘Gosh, I really do stand by my decision, and I really do want to go through with it.’ But the mandatory waiting period is there, and you -, you are trying to get on with your life, but ultimately there’s still something inside your body. So, that’s -, yeah, it keeps growing.”* (Naomi)

Christina, Gaelle and Angelique pointed to the abortion centres’ busy schedule, thwarting their desire for quick action:

*“I started making calls, but in centre A [abortion centre in city closest to participant’s home] I had to wait … for a long time (…) because they were very busy. Then I thought: ‘Gosh, I’m not going to be able to wait that long’, so I called centre B [abortion centre in city further away from participant’s home]. In centre B I could go a week earlier, or something like that. So, I opted for centre B then.”* (Christina)

*“The initial reason for that [choosing centre C] was because it could be done faster than at centre D. So, that was a difference of a week and a half.”* (Angelique)

As illustrated in the quotes above, the urgency felt by some women moved them to travel further in order to have an earlier abortion. Although “earlier” refers to a difference of one week for Christina and one and a half weeks for Angelique, it reflects the women’s perceptions of their situation as urgent and in need of immediate action.

### Grappling with blame and judgement

The majority of the participants perceived the experience of their unplanned pregnancy and the following abortion through the lens of self-blame. This self-blame was inextricably linked to the women’s expectation of social disapproval. To avoid potential judgement, participants were very cautious about disclosing their experiences with people in their social circle, “*because to this day, you still don’t know how people are going to react to it”* (Gaelle).

Most women spontaneously discussed feeling uneasy about themselves due to the later-in-life pregnancy and abortion. They labelled themselves as “dumb”, “stupid”, “unwise” or “naïve” for allowing an unplanned pregnancy to happen. Four women said that they felt ashamed, and two of them (Christina and Angelique) explicitly attributed this feeling of shame to their advanced age, indicating that they should have known better at their age:

*“I went to my GP whom I know well, and I was mortified of course. I was really embarrassed because, you know, you have enough contraception by now as not to have that happening at my age. It’s not very smart, is it?”* (Christina)

Irene and Juliet mainly expressed feeling ashamed at having had more than one abortion in their lives, and in the course of the interview they also lectured themselves, saying that they “*should have been more careful”*. In the quote below, Irene explained how “*history had repeated itself”* and how this had affected her self-image:

*“I don’t intend to go to that [abortion] centre a third time, you know. That would be completely ridiculous. I mean, I have two children, I’ve had two abortions, for all we know I had four daughters, you know. (…) So, I do have that image of myself now like: ‘Please, just do this properly for once, because these are things you can avoid.’”* (Irene)

Two participants (Irene and Christina) specifically referred to the unplanned pregnancy as “*their own fault”*. They identified the correct use of contraception as their responsibility as women. Christina was the only participant who became pregnant later in life by a man with whom she was not in a committed relationship. When she told this man about the pregnancy over the phone, she seemed to take all the blame, absolving him of any responsibility:

*“I said to him: ‘If you want to hang up the phone now, you have every right to do so’. Because like, yeah, it’s not … Women usually take the blame, like: ‘If only I had … ’ And I really took the blame on me. I’ve always had that actually, throughout the entire story. Like, he asked me: ‘Should I go with you [to the abortion centre]?’ But I said: ‘No’, I said, ‘you shouldn’t leave work to come to the centre with me’, like that. Because, like, I thought: ‘Imagine someone else having to make time to come along with me.’ (…) Yeah, I don’t know whether that’s right or wrong. It’s not okay, I thought afterwards. But then again, a man is not going to tell you, like: ‘I am as much at fault as you are’. Like, he didn’t say that either when I told him: ‘It’s my fault’. He didn’t say: ‘I disagree, we both did this’.”* (Christina)

Christina’s experience of how this man did not spontaneously acknowledge his share of responsibility for the pregnancy contributed towards her belief that it was her fault and her fault alone. Only Gaelle explicitly mentioned her partner’s co-responsibility for the unplanned pregnancy, framing the abortion experience as “*something they had to go through together”*.

Almost all participants framed abortion as a socially “sensitive and delicate” issue, with five women explicitly describing it as “taboo” in contemporary society. As most women anticipated negative reactions from people in their social circle, they typically shared their abortion experience with only two or three people, such as their sibling, best friend or close colleague, in addition to their partner. Three participants (Irene, Iris and Christina) did not disclose their abortion experience to anyone other than their partner (or, in Christina’s case, the man she had been casually dating). For most women, secrecy was the preferred strategy to avoid being judged and having “*miserably long discussions in which they have to explain themselves”* (Louisa). Some participants said that keeping quiet about their abortion was also a way of preserving social relationships (e.g. with colleagues with whom they have to work closely) or preventing people from seeing them in a bad light:

*“I wouldn’t dare say to someone, like: ‘You know what? I was pregnant’. If I were to say that to my colleague, for example, like, she would say: ‘But I told you so! Keep taking the pill. Come on!’ Because otherwise [if I told her], it’d make me feel … Can I say ‘vulgar’? I don’t want to come across like that, like: ‘Oh, yes, I was pregnant, but you know, I had an abortion for the second time. Whatever’. I don't want [other people getting] that idea.”* (Irene)

As some of the participants described an embodied experience of pregnancy (e.g. nausea, fatigue; see previous theme), they found it particularly challenging to keep their pregnancy secret. Nevertheless, some women considered sharing their experience with more people in the future, recognising the emotional benefits this could bring, such as feeling understood and being able to express their emotions freely, or because they felt the need to discuss the topic of abortion more openly, as Louisa stated: “*I want to show that not everyone should hide away”.* For two participants, one to two years had gone by after they had had their abortions. Over time, these women had shared their abortion experiences with several individuals whom they regarded as their “closest circle”. They demonstrated a sustained hesitation as they said that they would not engage in casual conversation about their abortion experiences “on a daily basis over coffee”.

Four participants expected unsupportive attitudes from professionals prior to arranging an abortion appointment. Christina recalled feeling “incredibly nervous” in her doctor’s waiting room, expecting her doctor to criticise her. However, all participants were positively surprised to receive nothing but supportive responses from professionals:

*“I had a good talk [at the abortion centre]. I shed a lot of tears, but the psychologist believed me completely, he was very supportive. He thought that what I had decided was normal. He said, like: ‘These seem to me to be all the right reasons to do this now’. So, that support was incredible.”* (Louisa)

This understanding attitude on the part of professionals reassured the women that they were not doing anything wrong and contributed significantly to how they coped with the abortion experience. Another way in which professionals sought to normalise the abortion experience was by providing women with statistics:

“They also gave figures in the [abortion] centre. The fact that as many women over 40 as under 20 have abortions** *gave me a more reassuring feeling. Like: ‘OK, you’d rather not do this’, but I was like, ‘I’m not the only one’.”* (Angelique)

These messages of normalisation of abortion provided the women with emotional support and reduced their feelings of shame.

## Discussion

This study offers insight into the lived experiences of women who had an abortion at the age of 40 or older. Previous research has suggested that age may be a relevant factor in abortion decisions.^11^ We specifically aimed to explore advanced reproductive age as a factor potentially guiding women’s abortion decision-making. We found that participants made the decision to terminate their later-in-life pregnancy quickly and often reaffirmed what they had previously agreed upon as a couple. This is in line with the findings of Rowlands^39^ that many women make hypothetical decisions about what they would do in the event of a positive pregnancy test. Although the women (and their partners) needed little time to make their decision, our findings demonstrate that they deliberately considered various factors. All women described a combination of factors that coincided and contributed to their abortion decision, such as family composition, relationship stability, reproductive desires and financial status. Age was cited by half of the participants and was primarily presented as an obvious reason to abort since the women assumed that childbearing at their age diverged from social norms and generally accepted timetables.^40^ However, age was never presented by these participants as the sole or most fundamental factor in their decision to have a later-in-life abortion. Instead, their accounts centred on their families, emphasising a desire to avoid family destabilisation and potential harm to its members through the choice of abortion. This resembles previous research on mothers (with an average age of 29.4 years) who had abortions to protect their existing children.^12^ The women in our study emphasised the importance of their romantic relationships, their children’s well-being, their own well-being, and the interconnectedness of these factors. They believed that abortion was the right choice not only for themselves but also for their family as a whole. Therefore, it is important to understand the reasons why women terminate their pregnancies within a comprehensive framework of normative conceptions about what constitutes a well-timed life course and individual and family-specific circumstances, desires and needs.^41^

Some participants in this study identified as Catholic. The Catholic Church and Christian political parties regularly adopt explicit restrictive stances on the issue of abortion in the Flemish media and through political debate. Nevertheless, we found that none of the participants regarded the views of the Roman Catholic Church as relevant to their decision to have an abortion, nor did they discuss religious considerations in relation to their beliefs about good mothering and appropriate reproductive timing. This should be understood within the Flemish cultural context. The majority of Flemish Catholics adhere to symbolic religiosity,^42^ meaning that they strive to maintain Christian traditions such as baptism, confirmation, and Christian burial, without actively engaging in regular religious practices or following Christian norms and guidelines.^43^ Similar findings have been presented in the context of reproductive decision-making in Flanders.^44^

Most participants found the discovery of the unplanned pregnancy and the abortion procedure emotionally distressing. Rather than being caught in a moral conflict about whether or not to have an abortion,^45^ our participants primarily felt an emotional burden due to their dissatisfaction with what they perceived as an avoidable and, for some, shameful event. To relieve themselves from psychological and physical strain, all participants promptly took action to secure an appointment at an abortion centre. Some were willing to travel to more distant locations in order to access abortion services sooner. This experience of urgency has also been described in a recent Australian study^46^ and appears to be largely unrelated to age. Delays caused by the mandatory waiting period and/or packed schedules at abortion centres added an emotional burden for a number of women in this study. These issues are interrelated, as the mandatory waiting period requires scheduling two appointments per woman. Research demonstrates that women often make the decision to have an abortion before consulting a healthcare professional,^8,10,47^ which is also supported by our findings. Mandatory waiting periods may perpetuate paternalistic tendencies that undermine women’s agency and reproductive autonomy.^48^ However, when professionals are involved, it remains important for them to consider each abortion seeker’s unique circumstances, to ensure their choice is fully autonomous, and to detect any potential coercion, making an individual case-by-case approach preferable.

Although previous research has suggested that younger or unmarried women may be more likely to (expect to) experience stigma in relation to both unplanned pregnancies and abortions,^49^ our findings indicate that advanced reproductive age does not necessarily reduce self-blame or expectations of social disapproval. For some women in our study their advanced reproductive age was an additional reason to blame themselves for the unplanned pregnancy as they believed they should have known better at this stage in their lives. Similar to younger women’s non-disclosure behaviour,^50^ our participants generally opted for secrecy to avoid negative judgment. Three participants chose not to share their abortion decision and experience with anyone other than their partner. In our study, other reasons for keeping the abortion a secret included preserving social relationships and preventing others from seeing the woman in a bad light, comparable to what Cockrill and Nack^12^ described as “maintaining a good reputation”. However, non-disclosure behaviours sustain abortion stigma^51^ and for many women it involves suppressed emotions and social isolation.^52^ Several participants considered sharing their abortion experience with more people in the future, citing potential benefits such as feeling understood, being able to express their emotions freely, and promoting open discussion of the topic. These findings underscore a need for destigmatisation of abortion for women of all reproductive ages. Informal platforms that allow for open conversations about abortion and the diverse circumstances in which abortion decisions are made can contribute to a general trend of increased understanding and normalisation of the topic. Public discourse plays a significant role in portraying women who choose to have an abortion as “normal women”,^53^ and in the social perception of abortion as an essential sexual and reproductive right.

### Strengths and limitations

This study’s sample and findings have several limitations. First, the sample lacks ethnic diversity. In 2023, 21% of the Belgian population had a foreign background, and 13.4% were non-Belgian.^54^ Even though the only inclusion criterion at the time of recruitment was being a woman who has opted for an abortion at an advanced reproductive age (i.e. 40 years or older), and information letters were provided in both Dutch and English, recruitment proved to be difficult. Second, although financial status was mentioned by a couple of participants as a contributing factor in their decision to have an abortion, the researchers did not collect any further information about the participants’ family income. As a result, no conclusions can be drawn about the socioeconomic status of the women and their families. However, it is possible that advanced-age women, on average, are more financially stable and/or have more flexible working hours than younger women. This may give them the resources (e.g. time, transport) to travel further to visit an abortion centre. Third, two participants were interviewed one to two years after their abortion. This may have interfered with their recall of events, including their decision-making process and emotional response relating to the abortion. Additionally, it is important to note that the sample consisted of only one woman who became pregnant later in life while casually dating, and it did not include advanced age women who have had abortions but have no children. Future research would benefit from examining the experiences and needs of women with different characteristics. The study’s strength lies in its focus on advanced reproductive age as a factor potentially guiding women’s decision-making and their experiences with abortion. This is a unique approach that has not been explored in recent qualitative studies on abortion. Furthermore, all interviews were conducted by an interviewer who was unaffiliated with the LUNA abortion centres, at a separate location. This allowed participants to openly share their abortion experiences and their perspectives on the care they received.

## Conclusion

This study’s findings indicate that women of advanced reproductive age who have children may consider age and life stage when deciding to have an abortion, but their primary concern is often their family’s well-being. Although the women felt confident in their decision to terminate the pregnancy, many experienced emotional distress and shame. For some, their advanced reproductive age intensified their feelings of self-blame for the unplanned pregnancy. Anticipating social disapproval, the participants typically chose to remain silent about their abortion experience despite recognising the emotional benefits of sharing their experience with people in their social circle. This study enhances our understanding of the experiences and meaning-making of abortion in women of advanced reproductive age and highlights some important implications for practice. We found that it is important for professionals and researchers to consider family preservation and advanced reproductive age as potential factors in shaping abortion decisions, and their connection to social norms about appropriately timed motherhood and family building. These findings also emphasise the need to destigmatise the topic of abortion in order to support women of all reproductive ages who have had an abortion.

## Data Availability

The data supporting the findings of this study are available from the corresponding author upon reasonable request. The data are not publicly available due to privacy restrictions.

## Footnotes

* We acknowledge that non-binary individuals and trans men may also have abortions. However, as the focus of this study was specifically on cisgender women, we have chosen for our terminology to reflect this.

† The necessity of the mandatory waiting period of 6 days is currently under review by Belgian legislators. At present, the waiting period can only be shortened or waived in cases of urgent medical situations.

** Official statistics for Belgium show that in 2021 6.2% of all abortions occurred in women aged 40 or older and 8.6% in women aged 19 or younger.
